# Statin Modulation of Human T-Cell Proliferation, IL-1*β* and IL-17 Production, and IFN-*γ* T Cell Expression: Synergy with Conventional Immunosuppressive Agents

**DOI:** 10.1155/2013/434586

**Published:** 2013-09-18

**Authors:** Ashmal Jameel, Kenneth G.-J. Ooi, Natasha R. Jeffs, Grazyna Galatowicz, Susan L. Lightman, Virginia L. Calder

**Affiliations:** ^1^UCL Institute of Ophthalmology, 11-43 Bath Street, London EC1V 9EL, UK; ^2^Moorfields Eye Hospital, London EC1V 2PD, UK

## Abstract

HMG-CoA reductase inhibitors (statins) have been demonstrated to be immunomodulatory for human immune-mediated disease and in experimental models. The aim of this study was to compare statin-mediated immunosuppressive effects on human T-cell responses *in vitro* with those of conventional immunosuppressives (dexamethasone, cyclosporin A (CsA), mycophenolate, and rapamycin). Statins (atorvastatin, lovastatin, and simvastatin) were investigated for their modulatory effects on human PBMC viability, cytokine profiles, and T-cell proliferation. At concentrations that inhibited anti-CD3/28-stimulated T-cell proliferation (*P* < 0.01), simvastatin significantly decreased intracellular CD4^+^ T-cell expression of IFN-*γ* (*P* < 0.01) to levels similar to those induced by conventional immunosuppressives. Atorvastatin and lovastatin also decreased IFN-*γ* expression, although to a lesser degree (*P* < 0.05). All three statins reduced levels of IL-17 production (*P* < 0.01). However, in response to anti-CD3/28 stimulation, simvastatin significantly upregulated IL-1*β* production (*P* < 0.05). The profile of cytokines produced in response to anti-CD3/28 stimulation was similar when both atorvastatin and dexamethasone were added as compared with dexamethasone alone, suggesting that atorvastatin can synergise with dexamethasone with respect to immunomodulation of cytokines. This data supports the hypothesis of selective statin-mediated immunomodulatory effects on human immune cells.

## 1. Introduction

As a therapy for hypercholesterolaemia, statins have been used clinically for over two decades. However, over the last decade, immunosuppressive effects have also been demonstrated which are independent of their cholesterol-lowering properties [1]. Statins *in vitro* modulate cell adhesion through effects on endothelial cells and leukocytes, via blocking activation of LFA-1 and decreasing ICAM-1 and MCP-1 expression on activated leukocytes and endothelium [2–4]. Statins have been shown *in vitro* and *in vivo* to reduce leukocyte motility, migration, and infiltration [5]. As compared to cyclosporine, statins were effective in reducing leukocyte infiltration in a rat model of allograft rejection [6]. They also inhibit the NF-*κ*B pathway, involved in transcriptional regulation of cytokines, chemokines, and adhesion molecules [7]. Statins have been shown to upregulate suppressor of cytokine secretion (SOCS) 3 and SOCS 7 which, in turn, downregulate IL-23 and IL-6 production, thus decreasing IL-17 production [8]. Clinical studies of statins in patients with immune-mediated diseases such as rheumatoid arthritis (RA), multiple sclerosis, and organ rejection following transplantation have shown conflicting results due to different statins being used in those studies and often open or retrospective studies involving small patient numbers [9, 10].

Immunomodulatory effects in experimental autoimmune encephalitis (EAE) have been observed with atorvastatin attenuating murine EAE [11], and it was found that atorvastatin upregulated IL-4, IL-5, and IL-10 and downregulated IL-2, IL-12, IFN-*γ*, and TNF-*α*. However, in another study investigating atorvastatin in murine experimental autoimmune uveitis (EAU), atorvastatin was found not to modulate the immune response despite histological grading suggesting mildly decreased inflammation [12], whereas in murine EAU attenuated by lovastatin, there was a decrease in IFN-*γ* production but no effect on Th2 cytokines [13]. In a Lewis rat model, EAU was decreased in severity by both atorvastatin and lovastatin, even when given after disease onset. With both of these statins, there were decreases in clinical and histological disease scores, antigen responsiveness, and IFN-*γ* production [14]. Simvastatin was reported to decrease cytokine production, including IL-10, in a murine model of collagen-induced arthritis [15] and in a murine lupus model, it decreased serum TNF-*α* and IFN-*γ* levels but increased transcription of IL-4 and IL-10 [16]. Overall, there appear to be a range of immune-related effects by statins depending on species, model, and cell type investigated.

Anti-inflammatory therapy for intraocular inflammation often requires use of corticosteroids, yet these can have severe side effects in the eye including raising intraocular pressure, cataracts, and glaucoma. To reduce these effects, steroid-sparing agents are also used including cyclosporin A (CsA), mycophenolate, and rapamycin. However, these drugs all have systemic side effects which limit their use in the long-term management of chronic disease. In chronic immune-mediated conditions such as RA and systemic lupus erythematosus (SLE), there is an associated increased premature atherogenesis and cardiovascular disease risk secondary to inflammatory processes [17–19]. In addition, patients with uveitis who are treated with steroids and immunosuppressive agents such as cyclosporine and mycophenolate have an increased risk of developing cardiac disease. Therefore, in these cases, the combined cholesterol-lowering and anti-inflammatory properties of statins may be clinically very useful.

The aim of this study was to determine whether individual statins exert immunosuppressive effects on T cells equivalent to those of conventional immunosuppressive agents *in vitro*. The effects of atorvastatin, lovastatin, and simvastatin on normal human whole blood-derived T-cell viability, proliferation, and cytokine responses were studied and compared to dexamethasone, CsA, mycophenolate, and rapamycin. The effects of combining atorvastatin with dexamethasone were also investigated.

## 2. Materials and Methods

All drugs were dissolved in either dimethylsulphoxide (DMSO), RPMI 1640 (Dutch Modification), and/or 100% ethanol. Atorvastatin (Parke-Davis, Pfizer Inc., NY, USA) was prepared as a 1 mM stock solution; lovastatin (Calbiochem, Nottingham, UK) at 40 mM; simvastatin (MSD, NJ, USA) at 10 *μ*M; dexamethasone (Chauvin, KingstonUpon-Thames, UK) at 400 *μ*g/L.; mevalonate (Sigma-Aldrich Company Ltd, Dorset, UK) at 0.5 M; mycophenolate mofetil (Roche Products Limited, Hertfordshire, UK) at 10 mM; rapamycin (Wyeth, Pfizer, Berkshire, UK) at 0.1 mg/mL; cyclosporin A (Sandoz, Novartis Pharmaceuticals, Surrey, UK) at 1 mg/mL.

### 2.1. Donors

Peripheral blood was obtained from 16 healthy donors (all working at the Institute/Moorfields) with informed consent (mean age (range), 34.3 (22.5–46.7) years; six males). Exclusion criteria included a history of autoimmune disease, atopy, haematological disorder, or current usage of systemic medication.

The protocols used in this study were reviewed and approved by the Local Ethics Committee. All studies involving human subjects were conducted according to the tenets of the Declaration of Helsinki.

### 2.2. Reagents

All tissue culture reagents were purchased from Sigma-Aldrich unless otherwise specified. All assays were performed in RPMI 1640 (Dutch Modification) supplemented with 2 mM L-glutamine, 10 *μ*g/mL gentamycin, 20 *μ*M 2-ME, nonessential amino acids, sodium pyruvate, and 10% human AB^+^ serum.

### 2.3. Proliferation and Viability Assays

Peripheral blood mononuclear cells (PBMC) were isolated as previously described [20]. Viability and proliferation assays were performed in triplicate. Cells were incubated for 10 min in a 37°C water bath with 7.5 *μ*M CFSE (Molecular Probes, UK) in serum-free medium. 1 mL cold stop buffer (10% FCS in RPMI) was then added before incubation at room temperature for 30 min. Cells were washed once with RPMI before being resuspended at 2 × 10^5^/mL in T-cell medium for 5 days with or without atorvastatin, lovastatin, simvastatin, rapamycin, mycophenolate, CsA, and dexamethasone. The reversibility of statin function was tested by addition of mevalonate [13]. T cells were stimulated with anti-CD3 (10 ng/mL; clone HIT3a) and anti-CD28 (5 ng/mL; clone CD28.2) antibodies (Pharmingen). All drugs or vehicle controls were added at start of culture, except CsA, which was added 2 h prior to stimulation since no effect was seen with CsA if added at time 0.

Two-color flow cytometry was performed (FACScan; Becton Dickinson, Oxford, UK; BD). Gates were set on viable lymphocytes according to forward (FSC) and side scatter (SSC). Listmode data was generated using CellQuest acquisition software on 15–25,000 events (BD). For viability assays, all lymphocytes were gated to assess level of nonviable propidium iodide expressing (PI^+^) cells. For proliferation, nonviable PI^+^ cells were excluded.

Data were analysed using WinList (Verity Software House, Topsham, ME). The total numbers of events were determined by analyzing the data using dot plots and rectangular regions to define the cell populations. Histograms were used to track the divisions of CFSE-labeled cells enabling identification of the percentage of divided (proliferated) cells. All data presented are from analyses of live (PI negative) cells only.

### 2.4. Cytokine Detection

Concentrations of statins required to achieve maximal inhibition of proliferation whilst maintaining viability were identified and used in a multiplex bead array to determine the effects of three statins on cytokine production. 100 *μ*L of heparinised whole blood was cultured with or without simvastatin, lovastatin, atorvastatin, dexamethasone, CsA, mycophenolate, and rapamycin and stimulated with PMA (50 ng/mL) and ionomycin (1 *μ*g/mL). Combinations of dexamethasone and atorvastatin were also included. Supernatants were harvested at 18 h, centrifuged to remove cells, and stored at −70°C. Multiplex bead cytokine arrays were conducted with a 10-plex kit (Bender Medsystems, Ebiosciences, Hatfield, UK) as per the manufacturer's instructions. Supernatants were analyzed simultaneously for IFN-*γ*, IL-1*β*, IL-2, IL-4, IL-5, IL-6, IL-8, IL-10, and TNF-*α*. The lowest levels of detection were IL-1*β* (6.2 pg/mL); IL-2 (6.3 pg/mL); IL-4 (5 pg/mL); IL-5 (5.8 pg/mL); IL-6 (14.8 pg/mL), IL-8 (58.6 pg/mL); IL-10 (7.3 pg/mL); IFN-*γ* (5.7 pg/mL), and TNF-*α* (8.1 pg/mL). IL-17A was assayed by ELISA (R&D Systems, Abingdon, UK), with a minimum level of detection of 62.5 pg/mL.

### 2.5. Intracellular Cytokine Expression

Whole blood cells were stimulated with PMA/ionomycin, and 10 *μ*g/mL Brefeldin A was added prior to culture for 18 h in the presence or absence of drugs. CD3^+^ T cells were stained intracellularly with mouse anti-human IFN-*γ*-FITC (clone 4S.B3) and rat anti-human IL-10-PE (clone JES3-9D7; both BD) as previously described [20].

### 2.6. Statistics

All intra-assay comparisons of means were analysed using Student's *t*-tests and for inter-assay data by Mann-Whitney *U* tests. Kruskal-Wallis tests were used for multiplex cytokine detection analysis for 6 donors, with nonparametric post hoc comparisons. In experiments with CsA, only 5 donors were included due to lack of available donors. Significance was reached when *P* < 0.05, and this was achieved for several assays, despite relatively small sample sizes.

## 3. Results

### 3.1. Effect on T-Cell Viability

**Figure 1:**
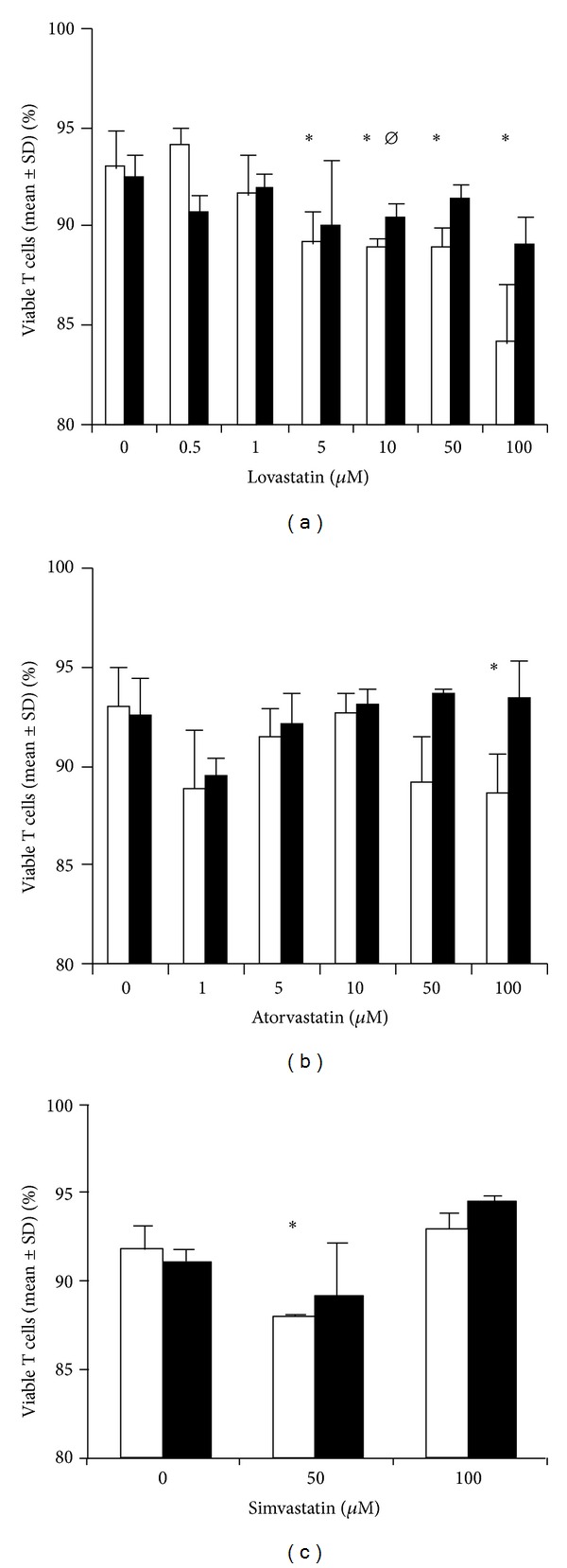
Cell viability was assessed at all concentrations of statins. The percentage viability is compared in the presence (filled bars) or absence of mevalonate (clear bars). ∗ denotes a statistical difference between viability of cells in different concentrations of statins (*P* < 0.05), and ⌀ indicates a difference in viability with and without mevalonate (*P* < 0.05).

To determine whether the drugs had any effect on T-cell viability, cells were cultured in the presence of drugs, mevalonate, and vehicle controls for 72 h prior to staining for PI (Figure 1). A range of concentrations were investigated, and viability was generally high with the lowest viability observed at the highest concentrations of lovastatin and atorvastatin. This was reversed with the addition of mevalonate, a downstream product of HMG-CoA reductase (*P* < 0.05) relative to lovastatin alone, suggesting that these higher drug concentrations reduced cell viability by inhibiting the mevalonate pathway and not via direct toxicity.

### 3.2. Effect on T-Cell Proliferation

**Figure 2:**
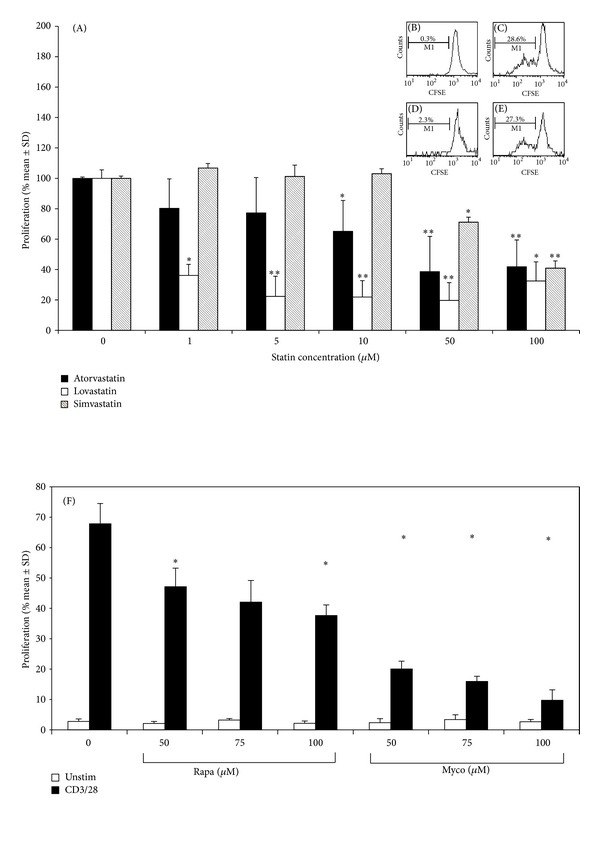
(A)–(F). Effect of statins on *α*CD3/*α*CD28-stimulated T-cell proliferation. Each data point represents the mean % T-cell proliferation ± SD. Due to intersample variation, data from each experiment (*n* ≥ 3) were normalized against the positive control of % proliferated T cells without drug, allowing experiments to be pooled (*n* ≥ 3). *P < 0.05 and **P < 0.01 as compared to control. Insert: one representative FACS analysis is shown. (A), Control histogram showing % divided T cells (M1) in the absence of stimulation or statin. (B), % divided T cells after stimulation but no statin. (C), % divided T cells with the addition of atorvastatin (50 *μ*M) as compared to stimulated control. (D), % divided T cells with the addition of atorvastatin (50 *μ*M) and mevalonate (200 *μ*M). (F), Effect of rapamycin and mycophenolate on *α*CD3/*α*CD28-stimulated T-cell proliferation. Each data point represents the mean % change in T-cell proliferation ± SD. Due to intersample variation, data from each experiment (*n* ≥ 3) were normalized against the positive control of % proliferated T cells without drug, allowing experiments to be pooled (*n* ≥ 3). *P < 0.05 and **P < 0.01 as compared to control.

There was a dose-dependent inhibition of T-cell proliferation with all statins, with maximal inhibition at 50 *μ*M atorvastatin and lovastatin and 100 *μ*M simvastatin (*P* < 0.01; Figure 2(A)). The addition of mevalonate, fully restored proliferation (Figures 2(B)–2(E) insert) confirming that these modulatory effects were statin-mediated.

A dose-dependent inhibition of anti-CD3/28-induced T-cell proliferation was also seen for the standard immunosuppressive agents with maximal inhibition at 100 *μ*M mycophenolate mofetil, 100 *μ*M rapamycin, (Figure 2(F)), 100 *μ*g/mL dexamethasone, and 100 ng/mL CsA (data not shown).

### 3.3. Effects of Statins on Cytokine Production

**Figure 3:**
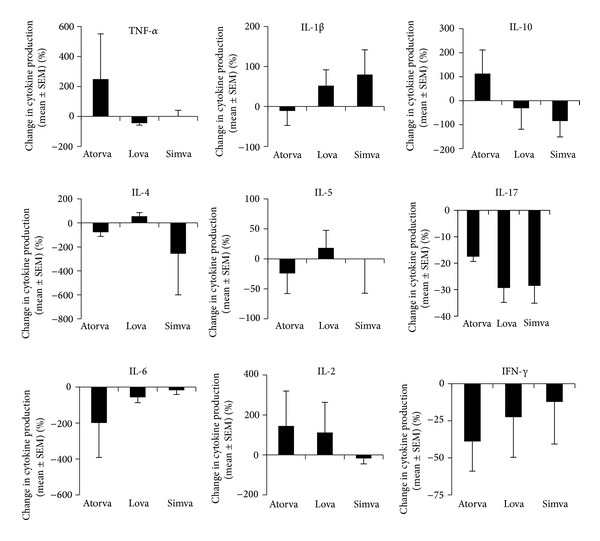
Graphs showing percentage changes in cytokine production by cells cultured with PMA/ionomycin ± statins. These studies show a multitude of varied responses indicating that the different statins have slightly different profiles of action.

The three statins demonstrated heterogeneous effects on cytokine production. Six human donors' PBMC were included in this study, and the responses were highly variable. The mean background levels of cytokines secreted by unstimulated cells were subtracted from all wells. The levels secreted by stimulated cells in the absence of exogenous drugs were as follows: TNF-*α* (30.31 ± 13.74 pg/mL), IFN-*γ* (0.66 ± 0.47 pg/mL), IL-10 (337.04 ± 374.8 pg/mL), IL-4 (4.47 ± 6.64 pg/mL), IL-1*β* (447.46 ± 236.07 pg/mL), IL-6 (2086.01 ± 489.49 pg/mL), IL-5 (14.53 ± 13.66 pg/mL), IL-17 (1186.50 ± 599.27), and IL-2 (1.50 ± 2.45 pg/mL). Following stimulation, there was variability in the cytokines produced among the donors, not unexpected in a mixed donor population. Nevertheless, for all six donors, simvastatin significantly increased IL-1*β* (Figure 3; *P* < 0.05), and all three statins inhibited IL-17 production (*P* < 0.01). Since not all donors' cells exhibited the same cytokine response profile, trends were observed although these failed to reach significance. All three statins caused decreases in levels of IFN-*γ* and IL-6. Atorvastatin reduced IL-1*β*, IL-4, IL-5, and IFN-*γ* and increased IL-10, TNF-*α*, and IL-2. Lovastatin increased IL-1*β* and IL-5 production and decreased IL-10, TNF-*α*, and IFN-*γ* production. Simvastatin was shown to decrease IL-2, IL-4, and IL-10 production. Mean levels of IFN-*γ* were relatively low, suggesting a low frequency of IFN-*γ* secreting cells in the normal donor population.

### 3.4. Differential Effects of Statins on Cytokine Responses

Due to the variable cytokine response profiles seen in individual donors, we investigated whether there were any correlations between the cytokines that were common to the drugs in comparison with cells stimulated in the absence of drug (PMA/ionomycin alone).  Table 1 summarizes the effects on cytokine profiles of the different drugs. All of the correlations included were statistically significant (*P* < 0.05). Stimulation in the absence of statins induced a strong correlation between IL-2 and IFN-*γ* and between IL-6 and TNF-*α*. In contrast, following atorvastatin treatment, IL-6 showed a good correlation with IL-2, IFN-*γ*, and TNF-*α*, and IL-2 correlated strongly with IFN-*γ* and TNF-*α*, as did IFN-*γ* with TNF-*α*. In the simvastatin-treated group, IL-6 correlated with IL-2, IFN-*γ*, and IL-1*β* and IL-2 with IFN-*γ*. Finally, the lovastatin-treated group showed correlations of IL-6 with IFN-*γ*, IL-5 with TNF-*α*, and IFN-*γ* with TNF-*α*.

### 3.5. Effects of Atorvastatin Combinations with Dexamethasone on Cytokine Responses

**Figure 4:**
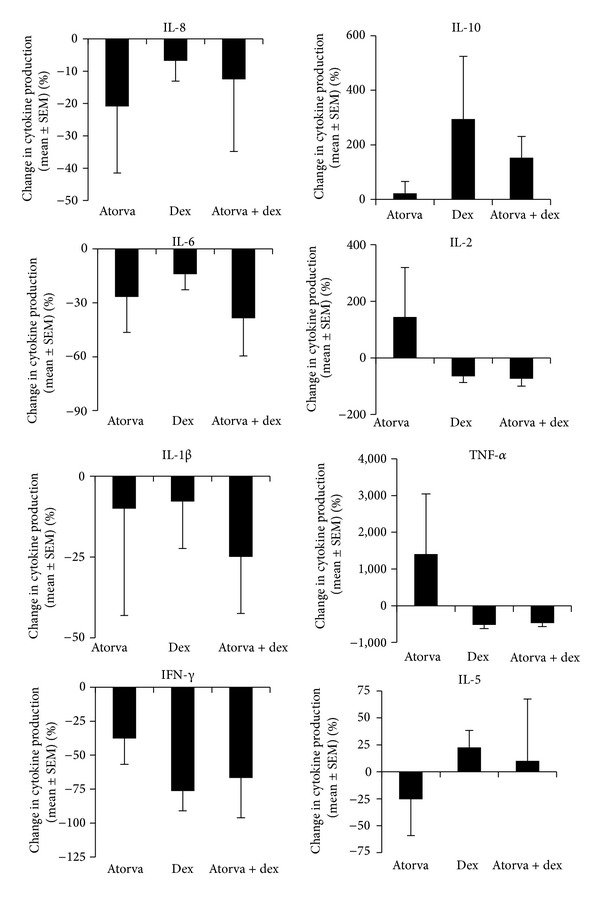
Graphs showing percentage changes in cytokine production in cells stimulated with PMA/ionomycin ± atorvastatin/dexamethasone.

Atorvastatin was selected to investigate its effects on cytokine responses in combination with dexamethasone. In the presence of either drug alone or when added in combination, IL-6, IL-8, IFN-*γ*, and IL-1*β* levels were all decreased although these did not reach significance, suggesting that the immunosuppressive effects of each of the drugs were maintained when added in combination (Figure 4). Interestingly, the level of IL-10 production was enhanced in the presence of dexamethasone, although this failed to reach significance. For the production of IL-2, IL-5, and TNF-*α*, the effects of atorvastatin were reversed upon addition of dexamethasone, or with dexamethasone alone, suggesting that this drug combination was antagonistic with respect to some cytokine responses. For the cytokine correlations (Table 1), there was a baseline correlation between IL-6 and TNF-*α* following addition of atorvastatin but not in the presence of dexamethasone alone or combined with atorvastatin. In contrast, the correlation of IL-2 with IFN-*γ* was maintained in the presence of either atorvastatin or dexamethasone alone but was lost when the drugs were added in combination. This suggests differential modulatory effects, with the combination of dexamethasone and atorvastatin exerting a distinct pattern of cytokine responses.

### 3.6. Effect of Immunosuppressive Drugs and Statins on Intracellular IFN-*γ* Expression

**Figure 5:**
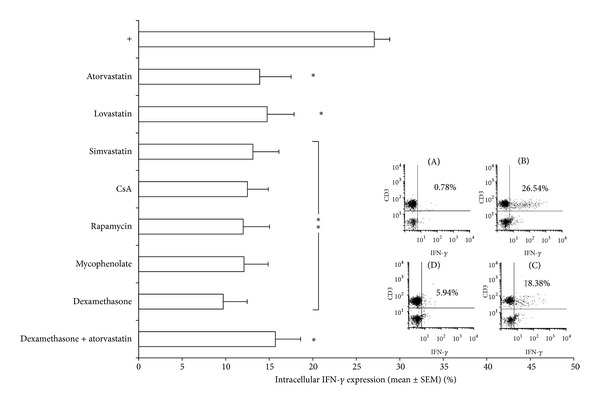
Percentage of IFN-*γ* expression in PMA/ionomycin stimulated whole blood with or without addition of drugs. Each bar represents the means ± SEM from 6 experiments, each performed in triplicate, subtracting basal cytokine expression. Drug concentrations: atorvastatin (50 *μ*M), lovastatin (50 *μ*M), simvastatin (100 *μ*M), rapamycin (100 *μ*M), mycophenolate (100 *μ*M), CsA (100 ng/mL), and dexamethasone (400 *μ*g/mL). *P < 0.05; **P < 0.01. One representative FACS analysis is shown. (A), Unstimulated CD3^+^ T cells showing basal IFN-*γ* expression. (B), Stimulated CD3^+^ T cells to demonstrate baseline levels of IFN-*γ* expression. (C), Stimulated CD3^+^ T cells treated with atorvastatin (50 *μ*M) demonstrating a decreased level of IFN-*γ* expression as compared to control. (D), Stimulated CD3^+^ T cells treated with dexamethasone (400 *µ*g/mL), showing a decreased level of IFN-*γ* as compared to group (C).

For a more sensitive method of detecting IFN-*γ*, intracellular cytokine staining was performed. There was a significant decrease in intracellular expression of IFN-*γ* in CD3^+^ T cells in the presence of the statins (*P* < 0.05 for atorvastatin and lovastatin; *P* < 0.01 for simvastatin). Dexamethasone, CsA, mycophenolate, and rapamycin also demonstrated significant decreases in intracellular IFN-*γ* expression as compared to stimulated controls (*P* < 0.01; Figure 5). Dexamethasone exerted the most significant reduction in expression of intracellular IFN-*γ*, whilst the combination of dexamethasone with atorvastatin also significantly reduced IFN-*γ* (*P* < 0.05).

## 4. Discussion

Statins have been demonstrated to exert anti-inflammatory effects *in vitro*: reduction in CD11b expression [21]; inhibition of IFN-*γ*-induced MHC-II expression on endothelial cells, macrophages, and therefore T-cell activation [22]; disruption of lipid rafts essential for T-cell activation [23]. Considering the use of statins as an adjunctive therapy for relapsing remitting multiple sclerosis, the 2010 Cochrane review on “Statins for multiple sclerosis” concluded that data from double-blinded randomized trials would be needed before licensing for treatment of multiple sclerosis [24]. In spite of the large amount of *in vitro* data indicating a potential anti-inflammatory role of statins, there is little evidence for their direct role in modulating human T-cell cytokines.

The concentrations of statins required to inhibit T-cell responses to anti-CD3/28 stimulation used for this *in vitro* study were selected from titration experiments, and these concentrations have been used in other *in vitro* studies [25–27]. Due to the potency of the *in vitro* stimulants, higher, nonphysiological concentrations of statins were required, and therefore, we cannot extrapolate the data to statin doses used clinically [28]. Nevertheless, these studies can inform on the effects of statins on human T-cell responses. Comparing the effects of drugs on T-cell proliferation, the statins inhibited proliferation to a comparable level to that achieved with conventional immunosuppressive drugs. The viability of the cells in our study was reduced in the presence of these statin concentrations, but, following the addition of mevalonate, there was a recovery both in viability and in proliferation, confirming that the inhibitory effect was reversible, and a direct result of statin-mediated inhibition of the HMG-CoA pathway.

Of the conventional immunosuppressive drugs investigated, dexamethasone exerted the most potent immunomodulatory effect, and its ability to decrease production of IL-1*β*, IL-2, IL-6, IL-8, IFN-*γ*, and TNF-*α* is in agreement with previous studies [29, 30]. Interestingly, dexamethasone treatment upregulated IL-10 production, as reported by others [31]. Of course the study design does not permit us to determine the cellular source of the cytokines, and monocytes, which were likely to be present at low numbers, have previously been observed to respond to simvastatin by upregulating IFN-*γ* production which, in turn, led to a decrease in IL-17 production [8]. The downregulatory effect of other immunosuppressive drugs (CsA, rapamycin, and mycophenolate) on IFN-*γ* expression is in agreement with other studies [29, 32–34].

These drugs also differentially inhibited other cytokines, with CsA significantly decreasing levels of IL-2, IL-4, and IL-5 IFN-*γ* but not IL-10 production (data not shown). In contrast, a decrease in IL-10 mRNA expression has been reported in a study on CsA-treated normal human whole blood following anti-CD3/CD28 T cell costimulation [29]. However, changes in IL-10 mRNA expression do not necessarily correlate with the level of protein production, which could explain the contradictory findings. Mycophenolate has recently been demonstrated to have a profound inhibitory effect on Th17 cells [35], and we have not yet investigated this drug for its effects on Th17 cells in our model (in progress). Other discrepancies in cytokine responses observed across the literature are easily explained by the different genetic backgrounds of donors used in different studies.

All three statins in this study significantly reduced intracellular IFN-*γ* expression, as previously reported for mouse and human T cells [15, 36, 37]. For the other cytokines investigated in this study, there were no effects common to all three statins. Intracellular expression levels of IL-2, IL-4, and IL-10 were not significantly altered (data not shown) due to the variable responses between the individual donors and very low levels of IL-4 and IL-10 expression.

Atorvastatin, when given to hypercholesterolemic patients, has been found to decrease PBMC production of TNF-*α* IL-1, IL-6 [38], IL-2, and IFN-*γ* *in vitro* [39]. Decreased serum IL-8 and IL-6 levels have been observed with atorvastatin postcoronary artery bypass grafts [40, 41]. More recently, atorvastatin has been reported to reduce the pathogenic production of IL-6 and IL-10 by activated T cells from SLE patients thereby readjusting them toward a more tolerant phenotype [42]. In our study, atorvastatin decreased production of IL-17 and intracellular expression of IFN-*γ*, in agreement with data from a Lewis rat model of experiment autoimmune neuritis [37] and in Balb/c mice with experimental colitis, in which TNF-*α* was also downregulated [43].

In murine EAE treated with lovastatin, reduced levels of IL-6, TNF-*α*, and IFN-*γ* with upregulation of IL-4, IL-5, and IL-10 were found, suggesting a Th2 polarization [44, 45]. We detected significant decreases in production of IL-17 and in IFN-*γ* expression in response to lovastatin.

Simvastatin decreased IL-17 and IFN-*γ* expression and increased IL-1*β*. It has previously been found that simvastatin decreased TNF-*α* in hypercholesterolemic patients [46] but had inconsistent effects on plasma IL-6 levels [47]. Simvastatin was not found to influence TNF-*α*, IL-6, and IL-1 receptor antagonist levels in an endotox in induced human *in vivo* model of low-grade inflammation [48] but decreased IL-8 production by PBMCs of CAD patients after 6 months of systemic treatment [49]. This contrasts with *in vitro* effects of simvastatin on anti-CD3/anti-CD28-stimulated PBMCs from RA patients with decreased IFN-*γ* and IL-10 unaffected [15]. Simvastatin also decreased levels of serum IL-6 in mouse collagen-induced arthritis [15] and IL-17 gene expression in human MS patients CD4^+^ T cells [8].

Due to the clinical use of atorvastatin for controlling cholesterol levels, it was selected in this study for possible combinatory effects when added with dexamethasone. However, by comparing atorvastatin, dexamethasone, and dexamethasone/atorvastatin together, no significant differences were found in overall levels of cytokines produced. However, the combination did affect cytokine correlations, suggesting that there might be an additive anti-inflammatory effect. There is little in the literature to suggest contraindications in terms of side effects, and the combination has been used experimentally in a rat model to reverse dexamethasone-induced hypertension [50].

In conclusion, different statins, with their distinct effects on cytokine responses, could have clinical applications in specifically targeting those key cytokines relevant to each inflammatory disease process, perhaps as a corticosteroid-sparing therapy, while providing effective disease control.

## Figures and Tables

**Table 1:** Spearman rank analysis of correlations in cytokines produced by cells cultured with PMA/ionomycin ± statins ± dexamethasone. The values represent the strength of the correlation of cytokines, with +1.00 being the maximum, and their changes in expression according to exposure to each statins. All results were statistically significant (*P* < 0.04).

	**No drug**		**Atorvastatin**			**Simvastatin**			**Lovastatin**			**Dex**		**Atorvastatin and dex**		
	IFN*γ*	TNF*α*	IL-2	IFN*γ*	TNF*α*	IL-1b	IL-2	IFN*γ*	IL-5	IFN*γ*	TNF*α*	IL-5	IFN*γ*	IL-1*β*	IFN*γ*	TNF*α*
IL-2	+0.89		■	+1.00	+0.99		■	+0.89				−0.94	+0.94			

IL-6		+0.89	+0.89	+0.89	+0.93	+0.94	+1.00	+0.89	+0.94	+0.89	+0.99			+0.94		

IFN*γ*	■			■	+0.99			■		■	+0.99	−0.89	■		■	

IL-10																+0.89

## Abbreviations

CsA: Cyclosporine A
HMG Co-A: 3-Hydroxy-3-methyl-glutaryl coenzyme A
EAE: Experimental autoimmune encephalitis
EAU: Experimental autoimmune uveitis
PI: Propidium iodide.
